# Pliable Cognitive MAC for Heterogeneous Adaptive Cognitive Radio Sensor Networks

**DOI:** 10.1371/journal.pone.0156880

**Published:** 2016-06-03

**Authors:** Mohammed Al-Medhwahi, Fazirulhisyam Hashim, Borhanuddin Mohd Ali, Aduwati Sali

**Affiliations:** Department of Computer and Communication Systems Engineering & Research Centre of Excellence for Wireless and Photonic Networks (WiPNET), Faculty of Engineering, University Putra Malaysia, Seri Kembangan, Selangor, Malaysia; Nankai University, CHINA

## Abstract

The rapid expansion of wireless monitoring and surveillance applications in several domains reinforces the trend of exploiting emerging technologies such as the cognitive radio. However, these technologies have to adjust their working concepts to consider the common characteristics of conventional wireless sensor networks (WSNs). The cognitive radio sensor network (CRSN), still an immature technology, has to deal with new networks that might have different types of data, traffic patterns, or quality of service (QoS) requirements. In this paper, we design and model a new cognitive radio-based medium access control (MAC) algorithm dealing with the heterogeneous nature of the developed networks in terms of either the traffic pattern or the required QoS for the node applications. The proposed algorithm decreases the consumed power on several fronts, provides satisfactory levels of latency and spectrum utilization with efficient scheduling, and manages the radio resources for various traffic conditions. An intensive performance evaluation is conducted to study the impact of key parameters such as the channel idle time length, node density, and the number of available channels. The performance evaluation of the proposed algorithm shows a better performance than the comparable protocols. Moreover, the results manifest that the proposed algorithm is suitable for real time monitoring applications.

## Introduction

Implementing a wireless sensor network (WSN) in new monitoring applications requires several improvements to overcome the problems degrading the system performance. Typically, the WSN consists of tens to thousands of small, low transmission range, low data rate, low power, and low cost units called sensor nodes that are deployed in a large-scale area. The WSN also includes one or more sink nodes that are responsible for collecting the data from the sensor nodes and delivering it to the data center or to the internet. The WSN operates in license-free industrial, scientific, and medical (ISM) radio spectrum bands along with technologies such as the bluetooth, cordless phones, remote control, and WiFi that share the same bands. The low capability of the sensor node is the primary obstacle against achieving a high performance in a congested radio environment with a high degree of contention caused by the radio spectrum scarcity. The limited capability of the traditional WSN node along with the hostile radio environment reinforces the trend of increasing the robustness of radio communications by exploiting developed technologies such as the cognitive radio (CR) [1][2].

The cognitive radio (CR) is an innovative technology for supporting dynamic spectrum access (DSA) that helps in addressing spectrum scarcity problem. In CR networks, the part of the radio spectrum allocated to the licensed users called the primary users (PUs) can be shared with other unlicensed users called the secondary users (SUs). The SU can utilize the spectrum only if the PU is absent, in an opportunistic manner because the later has a priority in the spectrum usage [3]. The cognitive radio sensor network (CRSN) is an emerging network that promises a wide spread usage of the wireless sensing applications. In the CRSN, some or all the nodes are equipped with radio and processing capabilities to perform an opportunistic and dynamic utilization of the spectrum holes in the licensed frequencies. Spectrum sensing (SS) tasks that are the main CR functions can be performed either centrally or in a distributed manner. The goal of the SS processes is to detect the presence or absence of the PU’s signal over one or more channels [4][5][6]. Although several detection techniques are available, energy detection (ED) is the most common. In ED, a channel is considered occupied if the detected signal exceeds a predefined threshold value.

Let *S*(*n*) denote the PU’s transmitted signal, and *H*_0_ and *H*_1_ indicate the absence of the PU signal or a channel idle state and the channel busy state or the presence of the PU signal, respectively. The detected signal can be expressed as,

$$\begin{array}{c} E\left(n\right)=\left\{\begin{array}{cc} W(n) & \;\text{,}\;H_{0}\\ W(n)+S(n) & \;\text{,}\;H_{1} \end{array}\right. \end{array}$$ (1)

where *W*(*n*) represents the additive white gaussian noise (AWGN). There are two important factors in the spectrum sensing process, namely the detection probability, *P*_*d*_ and the false alarm probability, *P*_*f*_. While *P*_*d*_ determines the probability that a channel is sensed as busy when it is actually busy, *P*_*f*_ determines the probability that a channel is detected as busy when it is free from the PU’s signal. The channel status is decided based on the comparison between the value of the received signal’s energy, *E* and the predefined threshold value, *T*:

$$\begin{array}{c} P_{d}=P_{r}\left\{D=1|H_{1}\right\}=P_{r}\left\{E>T|H_{1}\right\} \end{array}$$ (2)

$$\begin{array}{c} P_{f}=P_{r}\left\{D=1|H_{0}\right\}=P_{r}\left\{E>T|H_{0}\right\} \end{array}$$ (3)

Existing MAC algorithms for the conventional WSNs and the proposed MACs for the general cognitive radio networks (CRNs) are not suitable for CR-based WSNs. For instance, IEEE 802.15.4, the most common protocol in WSNs, does not meet the industrial network requirements because its main concern is to maintain low levels of power consumption throughout the duty cycle and it is designed for homogeneous types of networks. A homogeneous network is a network with only one application type [7]. Although IEEE 802.15.4e amendment and the other standards built on it such as the WirelessHART and the ISA-100.11a show certain advances in terms of the traffic variety, it is still not sufficient because a large amount of overhead data are produced and considerable power is consumed [8][9]. Meanwhile, the proposed algorithms for the CRN are also not appropriate for CRSN networks because these algorithms do not consider the resource constrained nature of the sensor nodes. Most studies focus on one aspect and are less concerned with the others. These aspects include the energy efficiency, latency decrease, interference avoidance, and an enhancement of the channel utilization.

Convinced of the fact that the fast and fine sensing operations adopted in IEEE 802.22 [10] cannot take place in the CRSN because they consume enormous power and require higher computational capabilities, some studies [11][12] deal with the energy efficiency concern in terms of the spectrum sensing processes only. Study [13] extends energy conservation to involve several fronts other than the developed spectrum sensing processes. Another study [14] offers real time spectrum allocation with a high utilization ratio, while other algorithms [15][16] aim to maximize the achievable throughput. Considering the application requirements, another study [17] introduces a dynamic resource management scheme to enable the CR network to coordinate the spectrum decision adaptively, dependent on the time-varying spectrum resources.

Considering that the common SS techniques do not assure the required levels of the signal detection performance, the federal communication commission (FCC) in the United States and the European communications committee (ECC) suggest the usage of a combination of solutions instead of depending on the SS operations only [18][19]. The proposed algorithm in this study, i.e. the pliable cognitive MAC (PCMAC), depends on a combination of database and SS techniques. This combination aims at enhancing the opportunistic utilization performance by protecting both the PU and the SU data from interference. The main contributions of this study can be summarized in the following statements:

- Proposing a new MAC algorithm for CRSN networks achieves the following:
  - A balance between the common features of the traditional WSN and the new requirements of the rapidly evolving new technologies.
  - Efficient exploitation of the CR technology to achieve the end-to-end objectives.
  - Coping with the styles of wireless networks that encompass new concepts such as the internet of things (IoT) and the machine-to-machine(M2M), with heterogeneity as one of their main features.
  - Efficient scheduling and resource allocation schemes for the intra-heterogeneous CRSN network.
- Modeling the performance of the proposed algorithm.
- Comparing the performance of the proposed algorithm with a comparable protocol.

This study presents a feasible CRSN MAC algorithm that considers the conventional sensor node characteristics. Significant computational complexity is not required because the main changes will be made in a limited number of nodes only, the master nodes, that will help mitigate the consumed power required in cognitive radio processes. Further, adopting a strategy of distributed spectrum sensing and centralized allocation decision-making eliminates collision problems that cause power wastage. Thus far, this paper is one amongst the few works that study the scheduling and resource management in CRSN networks with nodes that might contain either several types of sensor or several importance levels for the data.

The remainder of this paper is organized as follows: Section II reviews the related works. Section III presents the adopted network architecture for the proposed work. Section IV demonstrates the PCMAC. Section V shows the analytical modeling for the most important performance metrics and Section VI presents the PCMAC evaluation results. The last section is the conclusion.

## Materials and Methods

### Related Works

Cognitive adaptive MAC (CAMAC) algorithm, which is carrier sense multiple access (CSMA)-based, proposed in [13] for the ad hoc-based CRSN targets unlicensed ISM bands and deals with the IEEE 802.11 users as the primary users. The main concern of the proposed algorithm is to reduce the power consumption and to do so it limits the SS operations to on-demand operations. The CAMAC protocol consists of three phases: spectrum measurement, negotiation and contention, and data transmission. In the first phase, the predefined nodes perform the spectrum sensing measurements and nodes that desire to send data have to contend to access the common control (CC) channel through a slotted ALOHA-based technique. These nodes have to contend again in the data channels to submit their data using a CSMA-based technique. This protocol is designed for homogeneous networks and is not event-driven; thus, it cannot cope with the common and emerging applications. Further, this protocol lacks efficient duty cycling because the nodes have to be awake even if it is unnecessary. This protocol also induces a significant overhearing problem.

A previous study [20] enhances and analyzes the performance of the dynamic open spectrum sharing (DOSS) MAC protocol that is CSMA-based and incorporates a multiple channel access. The targeted topology is ad hoc-based and the elected performance metrics are the delay and the throughput. The DOSS MAC protocol uses a common control channel to facilitate negotiations between the SUs, i.e. the sensor nodes, regarding the management of the data traffic channels. The sensor nodes, according to this algorithm, have to perform at least three spectrum sensing operations to submit a data packet to a receiver node in a multi-hop scheme. A high probability of collision is yield because the SS decision making is distributed. Consequently, several sensing operations are required because the probability of making similar decisions is high between the adjacent nodes. Moreover, a long idle listening time for the nodes, for remaining updated about the occupied channels, becomes inevitable and the consumed power increases. The delay model is based on an M/G/C priority queue though the system uses a common control channel that is based on carrier sense multiple access with collision avoidance (CSMA/CA) scheme. The prioritization is inter-network, i.e., between different networks, where the PU network users have the highest priority. In the bandwidth model, the study assumes that a successful transmission might occur even if it encounters an interference case between the PU and the SU signals that explains the high achieved throughput. Likewise, the study [21] applies the M/D/1 priority queueing scheme to model the delay. A delay analysis of the opportunistic access in a time-slotted CR-based system is performed. The adopted model considers a network with one primary user and one secondary user. The link they share is slotted and the SU has to sense the medium in the beginning of each slot to ensure that it is not occupied by a PU transmission.

Another study [14] proposes a dynamic open spectrum sharing MAC protocol for wireless ad hoc networks. This protocol aims to offer real time spectrum allocation with a high utilization ratio and to overcome the hidden terminal problem. The network model is ad hoc-based with a common control channel. The protocol uses a cyclostationary detection technique for the presence of the PU signal as the first of the five steps of the protocol. Each SU should have at least two transceivers to deal with the complex work flow, in particular, those that are related to the physical layer functions and the spectrum negotiations. On the contrary, in another work [22], the proposed algorithm is cluster-based and the cluster head not only manages the channel assignment but performs the spectrum sensing operations as well. The proposed slotted MAC enables the node to monitor the PU’s behavior during the transmission to handoff the channel if a PU presence is detected. A framework is proposed in [16] to maximize the gathered data and increase the lifetime in a multi-hop CRSN. Similarly, the proposed algorithm in [15] and its modeling of the opportunistic spectrum access is CSMA/CA-based in the time slot’s contention phase. The achieved throughput can be significant because the winning SU utilizes the entire available bandwidth; however, the PUs are strictly synchronized and an increased complexity is required to manage the reporting process in the case of a high SU density. In this work, the delay and throughput metrics are modeled. Another work [23] aims to maximize the network lifetime by efficiently alternating the active mode and the sleep mode; the scheduling problem is assumed to be nondeterministic polynomial time (NP-complete). The proposed algorithm is based on dividing the nodes into sets according to the obtained values for the detection and false alarm probabilities for each node.

Instead of the power efficiency, the design and performance analysis in [24] is based on the IEEE 802.15.4 MAC protocol to address the QoS for both the constant bit rate (CBR) traffic and the best effort (BE) traffic. The proposed work does not consider the significant power consumption caused by the considerable idle listening time. Moreover, the protocol lacks an efficient resource allocation scheme and a prioritization scheme among the different nodes. Generally, the proposed works are ad hoc-based and are less efficient in dealing with the application QoS requirements or the traffic patterns.

## Pliable Cognitive MAC (PCMAC)

### Network Model

By employing the concept of business continuity (BC), the adaptive CRSN transforms its operating architecture and working model into a CR-based architecture. The adaptive CRSN aims to avoid a significant degradation of the network performance or achieve an acceptable performance level in case of a network failure. This network model attempts to maintain the conventional WSN characteristics and cope with the required QoS for emerging wireless sensor applications. Moreover, this architecture promotes the idea of employing a new technology that utilizes the cognitive radio, while maintaining the common characteristics of the conventional WSN. Additionally, the cluster-based topology is more appropriate for heterogeneous networks than the ad hoc-based topology because it handles the scalability issue well and mitigates the effects of the idle listening problem that helps in decreasing the consumed power [9][25]. The transition cases from the normal network model to the abnormal model include network failure, significant performance degradation, and special transmissions. Thus, ideal sensor nodes have two ways of transmitting data: a normal transmission where the sensor node transmits data to its cluster head (CH) node; and an abnormal transition where the sensor node transmits data to the master node that in its turn forwards them to the sink node using CR techniques through a licensed frequency.

**Fig 1:**
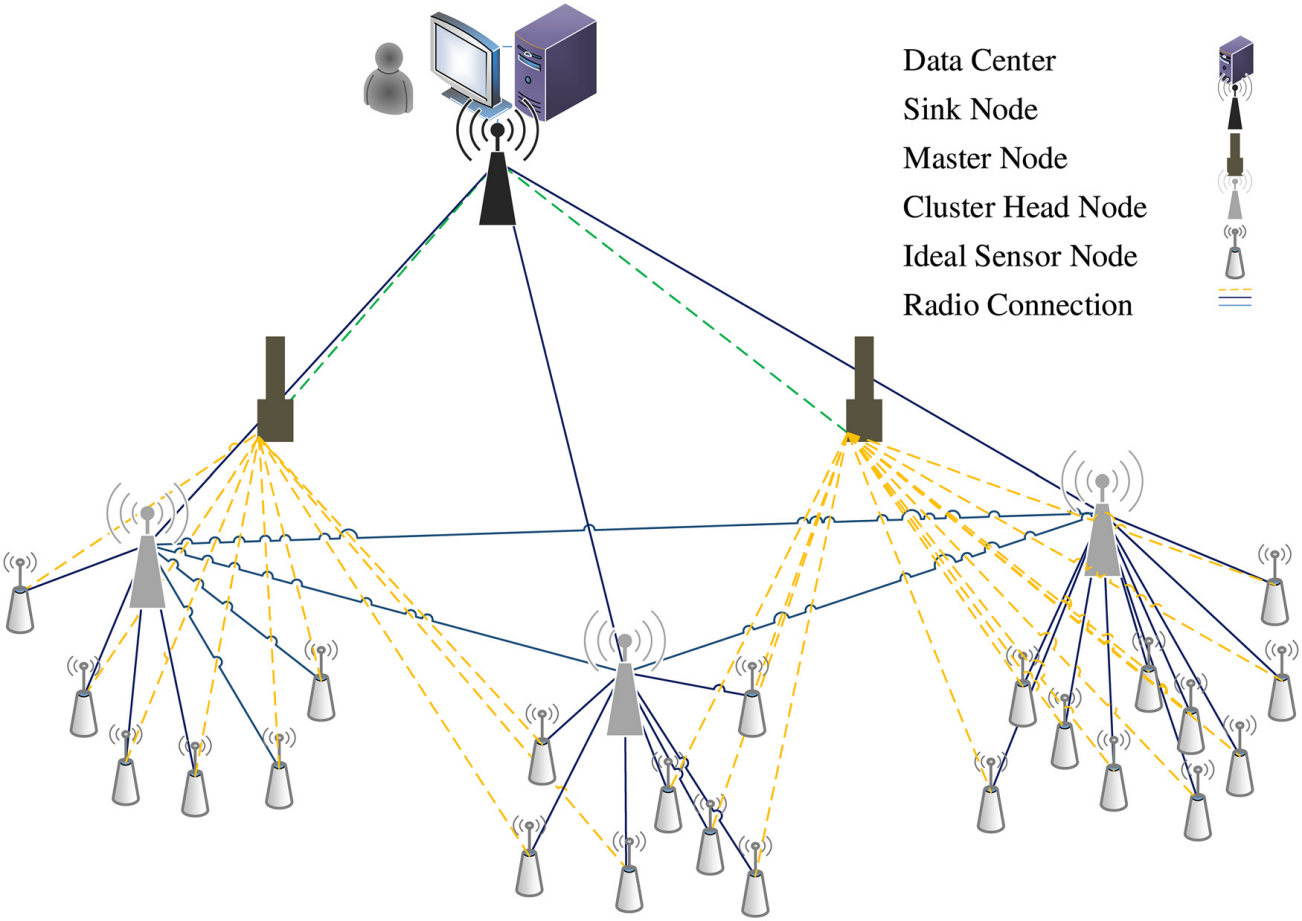
The adaptive architecture of CRSN.

The physical structure of the sensor nodes will not be changed and the power consumption will be enhanced because the ratio of the successful submissions will increase. Further, the ratio of the collision, considered as the main reason for the power wastage, will be reduced significantly. The master node acts as a relay node as it collects the sensor node data and forwards it to the sink node as well as performing several CR functions. The sink node is assumed to have *C* transceivers that can operate concurrently, while each master node has only two transceivers: one to communicate with the sensor nodes using the ISM band and the other to communicate with the sink node using the common control channel or the licensed channels. The proposed network model shown in Fig 1 assumes a heterogeneity in the network, either in the sensor’s application or in the traffic patterns. Considering the network’s heterogeneous nature during the design ensures the provision of the QoS for different applications. Practically, this consideration can be revealed in the adopted scheduling and in the resource allocation schemes that can be either traffic-based or node-based.

The traffic-based category can be divided into two main subcategories: criticality-based traffic and pattern-based traffic. In criticality-based traffic, data can be classified into safety, control, and normal data, whereas, data in the pattern-based traffic can be classified into a constant bit rate (CBR) traffic and burst traffic. The second category is node-based and encompasses the remaining energy-based, buffer size-based, and spatial position-based subcategories. In the proposed model, the scheduling and resource allocation schemes are based on the traffic criticality. Thus, a single ideal sensor node can send its packets to the master node with multiple levels of QoS requirements, with latency as one of the main. Each submitted packet is identified with a pattern that indicates its priority, enabling the master node to distinguish the packet type and to queue it accordingly.

### Proposed Algorithm

Communication between the sensor nodes and the master nodes is non-opportunistic, i.e., in the ISM bands, whereas, data communication between the master nodes and the sink node is opportunistic. As the entire traffic will be collected by the sink node, analyzing the channel characteristics via the statistics of the performed transmissions will be more beneficial if it is carried out by the sink node. Therefore, the proposed algorithm employs centralized decision-making and distributed spectrum sensing manners in radio cognition. The sink node performs the allocation decision-making for the channels in the targeted spectrum band. The sink node has an updated database of the allowed licensed channel that can be utilized opportunistically and dynamically. The database retains the status of each channel to determine if it is currently allocated to any master node and if it is suitable for transmission, depending upon the physical characteristics. Consequently, the master nodes perform the spectrum sensing processes based on the decision of the sink node. As a result of employing the aforementioned methods, periodic and redundant spectrum sensing operations that are not on-demand are not required, significantly enabling energy conservation.

Upon receiving traffic from the sensor nodes, the master node uses the common control channel to send its submission request with a request to send (RTS) message that also contains information about the status of the buffer and the importance level of the data. Within the master node buffer, packets are virtually queued in two queues according to the classification of the packets or the originating nodes. In each queue, the first come first served (FCFS) discipline is adopted for packet scheduling. The first queue contains the data of nodes that are more delay sensitive and more critical, having a high priority to reach the destination with a lower delay time; the packets in the second queue are more delay-tolerant. For example, in e-Health applications, the first queue represents the patient activity monitoring data such as the heart and brain monitoring, and critical alarms of the medical equipment. The second queue represents the normal monitoring traffic such as heart beat, blood pressure, and environmental measurements.

**Fig 2:**
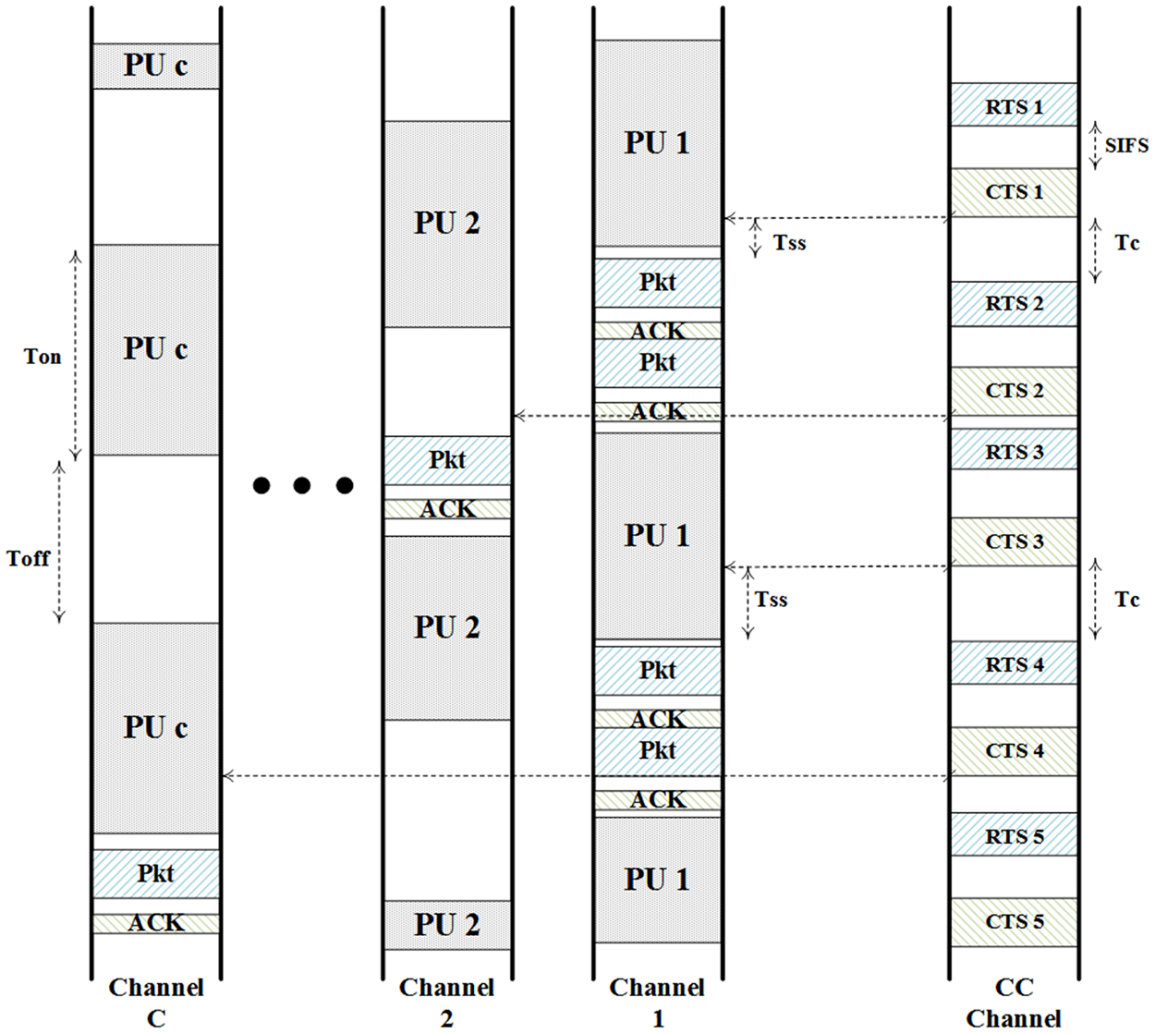
Spectrum access driven by the CSMA/CA-based common control channel.

The medium access scheme in the common control channel from IEEE 802.11 that is CSMA/CA-based is improved. Thus, the master node is forced to contend with the other master nodes to access the medium of the common control channel to send its request. The master node begins to sense the CC channel medium through clear channel assessment (CCA) operations; if it finds the medium free, it performs the RTS/CTS handshake. Otherwise, the master node backs-off for a while and senses the medium again. When the sink node receives the RTS message, it evaluates the information enclosed within the request and allocates the appropriate channel for the current packets’ submission, requested by the master node. The sink node responds with a clear to send (CTS) message enclosing information regarding the allocated channel. The targeted radio environment is assumed to be homogeneous, indicating that the included channels have similar spectrum hole lengths, physical characteristics such as the SNR, and primary user behavior. Thus, the sink node considers only the current allocation status of the channel. Both the sink node and the master node tune their transceivers to the elected frequency, and the master node continues sensing the channel until it finds the channel idle. Considering the Nyquist-Shannon sampling theorem in the sensing process, the sampling rate should be at least twice the bandwidth of the PU’s signal, i.e., *f*_*s*_ ≥ 2*W*_*PU*_, to ensure a high accuracy. Note that a higher sampling rate will result in an increase in the memory requirements and an excessive energy consumption not only in the sampling process, but also in the following processes such as the analog to digital conversion (ADC) and the signal processing. The master node will start transmitting the packet and when the sink node receives the submitted packet successfully, it responds with an acknowledgement (ACK) message for confirmation. Otherwise, the submission process is considered as failed and the master node tunes its transceiver into the common channel and contends to send an RTS request, again. The data transmission is in a burst-based manner implying that the master node will continue the transmission until either its buffer becomes clear of packets or it does not receives an ACK message for the last transmitted packet. As illustrated in Fig 2, regarding the enclosed information within the CTS 1 message, the master node begins to sense the allocated channel, channel 1, where a time, *T*_*ss*_, occurs until the channel is found free from the PU 1 signal. Once the channel is found idle, the master node starts to send its packets one-by-one and receives the acknowledgements subsequently.

**The algorithm simple pseudo code is as follows**:

1: *sort* data packets in the buffer of the master node according to their priorities

2: **for** any packet in the master node’s buffer **do**

3:  **repeat**

4:   *sense* the CC channel

5:   *backoff*

6:  **until** *S*_*rc*_ < *S*_*Tc*_ //*the received signal energy is less than a threshold value: the medium is free*

7:   *send* RTS message

8:   *electing* an unallocated channel among *C* channels //*by the sink node*

9:   *receive* CTS message

10:    *tune* the transceiver to the elected frequency

11:   **repeat**

12:    *sense* data channel

13:   **until** *S*_*rd*_ < *S*_*Td*_//*the received signal energy is less than a threshold value: channel’s idle time*

14:    **repeat**

15:    *send* data packet

16:    *receive* ACK message

17:    **until** no packet to send or no ACK message

18: **end for**

For an optimal performance, the master nodes can be divided into sets of up to *N*_*s*_ nodes and the allocated channels to be utilized for each set are given as, *C*_*s*_. While deploying the master nodes, spatial correlation must be considered to enable the common control channel to be reused. The pliability of this algorithm is implied by the nature of the scheduling and resource management scheme. The pliability is based on a non-preemptive priority discipline in addition to the lack of complexity in the resource allocation between the nodes that ensure collision-free transmissions.

In addition to the scope of the study [20] mentioned previously, the proposed algorithm, in this study, decreases the minimum number of SS processes significantly. The PCMAC alleviates the idle listening effects and presents a more robust SS and decision-making scheme. Further, as shown in the next section, this study presents a more appropriate modeling scheme because it considers the common control channel as a mediator between the transmitted nodes and the data channels. Although the model chosen is M/G/1-based, it takes into account the fact that both the CC channel and the data channels share serving data packets.

### Algorithm Evaluation

To evaluate the proposed algorithm, two of the most important application performance metrics were modeled, namely, the average delay and the throughput. The delay performance indicates if the algorithm presents durable latency measurements and whether the bounds of the performance are acceptable. Meanwhile, the throughput evaluation helps in managing the radio resources efficiently. The effects of the main parameters, namely, the packet arrival rate, *λ*, the nodes density, *N*, the number of channels, *C*, and the channel’s idle time length, *τ*_*off*_, are studied. The study illustrates the effect of changing these parameters on the two metrics. The blocking probability in the common control channel is assumed to be zero because of the nature of the adopted scheduling scheme. Thus, a failed transmitted packet is sorted again in its queue for the next submission, regardless of its virtual queue. Consequently, utmost reliability is achieved for all the packets.

**Fig 3:**
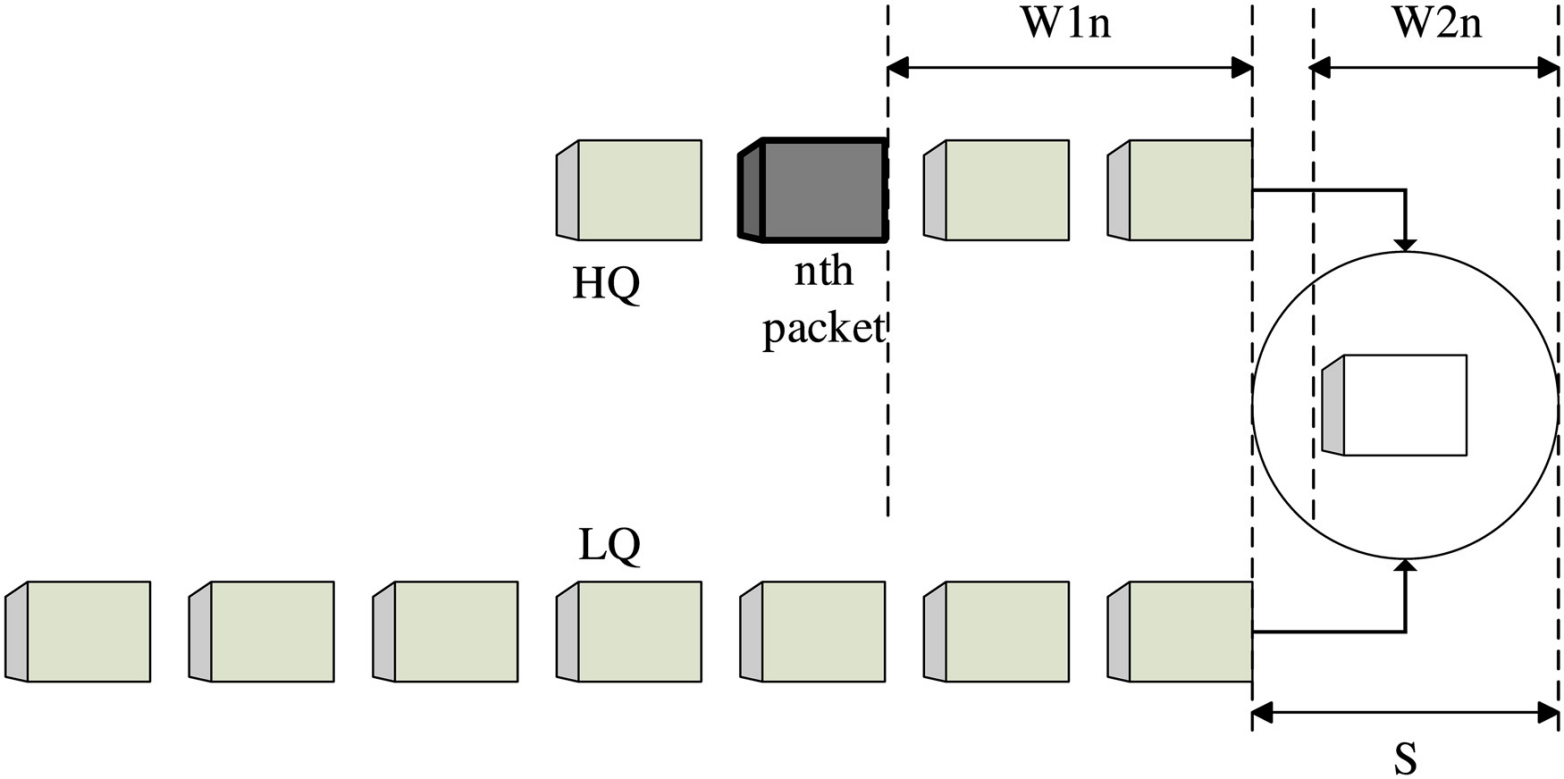
Virtual queues for packets.

An enhanced M/G/1 priority queuing is used to model the average delay, where M stands for Markovian, i.e., the system has exponentially distributed intervals for the arrival packets. G stands for the general distribution of the service time, and 1 represents the used server, CC channel. In addition to the server employed to serve the data packets in the proposed algorithm, the licensed data channels also contribute in this mission. The enhanced non-preemptive M/G/1 queue model has two virtual queues: a high queue (HQ) and a low queue (LQ). However, the HQ packets are prioritized, i.e., scheduled first, these packets will not interrupt the current service for the LQ packets. All the packets are assumed to have the same length. The end-to-end average delay time consists of two parts: the waiting time and the service time, S, where the waiting time for a packet in the master node consists of two parts, *W*1 and *W*2. While *W*1 represents the waiting time caused by the preceding packets in the queue, *W*2 denotes the waiting time because of a current submission from the same node even though it belongs to the other queue, as illustrated in Fig 3. The service time is estimated as

$$\begin{array}{c} S=T_{c}+T_{ss}+T_{tr} \end{array}$$ (4)

where *T*_*c*_ represents the contention time in the CC channel, *T*_*ss*_ is the consumed time in the data channel’s sensing processes, and *T*_*tr*_ is the packet’s transmission time. Based on Fig 2,

$$\begin{array}{c} {\bar{T}}_{c}=DIFS+{\bar{T}}_{b}+T_{RTS}+SIFS+T_{CTS} \end{array}$$ (5)

where DIFS and SIFS are the distributed and the short inter-frame spaces, respectively. While *T*_*RTS*_ represents the time at which an RTS message is sent, *T*_*CTS*_ denotes the time of the CTS message. A previously presented expression in [26] is used to estimate the mean backoff time as,

$$\begin{array}{c} {\bar{T}}_{b}=CW_{min}\frac{1-p-2^{N}p^{N+1}}{2-4p}-\frac{1}{2} \end{array}$$ (6)

where *CW*_*min*_ is the minimum contention window size, *p* represents the conditional collision probability, and *N* is the number of contender nodes. In the steady state,

$$\begin{array}{c} p=1-{\left(1-\tau \right)}^{N-1} \end{array}$$ (7)

where *τ* is the probability that the node transmits in a random time slot. For simplicity, based on the previously mentioned assumption [27],

$$\begin{array}{c} \tau =\frac{2}{CW_{min}+1} \end{array}$$ (8)

Meanwhile, the mean time estimated for a persistent spectrum observation is,

$$\begin{array}{c} {\bar{T}}_{ss}=\frac{\tau_{on}}{2}P_{on} \end{array}$$ (9)

*P*_*on*_ is the licensed channel occupancy probability (by a PU)

$$\begin{array}{c} P_{on}=\frac{\tau_{on}}{\tau_{on}+\tau_{off}} \end{array}$$ (10)

While *τ*_*on*_ represents the period where the channel is busy because of the PU’s transmission, *τ*_*off*_ expresses the idle channel period. In the proposed model, an accurate SS is assumed. Thus, *P*_*d*_ ≈1 and *P*_*f*_ ≈0.

As all the packets are assumed to have the same length, the estimated time for a packet transmission is,

$$\begin{array}{c} T_{tr}=CCA+\frac{L}{D_{lc}}+SIFS+T_{ACK} \end{array}$$ (11)

where *CCA* denotes the clear channel assessment mid time, *L* is the packet length and *D*_*lc*_ is the data channel’s data rate. According to Eq 4, the mean service time is,

$$\begin{array}{c} \bar{S}={\bar{T}}_{c}+{\bar{T}}_{ss}+T_{tr} \end{array}$$ (12)

The arrival of the packets in each virtual queue is assumed to be a Poisson stream; *λ*_*HQ*_ and *λ*_*LQ*_ represent the arrival rates of the HQ and LQ, respectively. The occupation rate of the common control channel is the sum of the occupation rates for both the queues, given as,

$$\begin{array}{c} \rho_{C}=\rho_{HC}+\rho_{LC} \end{array}$$ (13)

where

$$\begin{array}{c} \rho_{HC}=\lambda_{HQ}{\bar{T}}_{c} \end{array}$$ (14)

and

$$\rho_{LC}=\lambda_{LQ}{\bar{T}}_{c}$$ (15)

To maintain the system stability, the occupation rate for the common control channel must be less than one. Similarly, the occupation rate of the traffic channels after considering the number of utilized channels, can be estimated as,

$$\begin{array}{c} \rho_{HD}=N\lambda_{HQ}\frac{{\bar{T}}_{ss}+T_{tr}}{C} \end{array}$$ (16)

$$\begin{array}{c} \rho_{LD}=N\lambda_{LQ}\frac{{\bar{T}}_{ss}+T_{tr}}{C} \end{array}$$ (17)

It is also assumed that the duty cycle time is 1 s. The mean waiting time for a packet from the HQ is estimated as,

$$\begin{array}{c} {\bar{W}}_{HQ}={\bar{L}}_{HQ}\bar{S}+\rho \bar{R} \end{array}$$ (18)

where $\bar{R}$ is the mean residual service time of a current served packet, even it belongs to the LQ; *ρ* represents the general occupation rate of the entire traffic in the CC and the data channels. The model is non-preemptive. Thus,

$$\begin{array}{c} \bar{R}=\frac{{\bar{S}}^{2}}{2\bar{S}}=\frac{\sigma_{S}^{2}+{\bar{S}}^{2}}{2\bar{S}} \end{array}$$ (19)

By using Little’s law and when ${\bar{L}}_{HQ}$ represents the mean length of the HQ,

$$\begin{array}{c} {\bar{L}}_{HQ}=\lambda_{HQ}{\bar{W}}_{HQ} \end{array}$$ (20)

This equation can be written as,

$$\begin{array}{c} {\bar{W}}_{HQ}\left(1-\rho_{H}\right)=\rho \bar{R} \end{array}$$ (21)

where, *ρ*_*H*_ represents the sum of the HQ packets’ occupation rate in the control channel and the data channels. Thus, the mean waiting time for a packet from the HQ is estimated as,

$$\begin{array}{c} {\bar{W}}_{HQ}=\frac{\rho \bar{R}}{1-\rho_{H}} \end{array}$$ (22)

Therefore, the mean throughput time, i.e., the average delay time, is,

$$\begin{array}{c} T_{HQ}={\bar{W}}_{HQ}+\bar{S} \end{array}$$ (23)

Considering that the new packet may arrive at any time in an ongoing service with a mean service time, $\bar{S}$, the mean residual service time can be estimated as,

$$\begin{array}{c} \bar{R}=\frac{1}{2}\left(c_{s}^{2}+1\right)\bar{S} \end{array}$$ (24)

where *c*_*s*_ is the coefficient of the service variation time and is equal to *σ*_*s*_/*S*. Note that in case of an exponential service time, $c_{s}^{2}$ is equal to 1 and in case of a deterministic service time it equals 0. For simplicity, $c_{s}^{2}=0.5$. Thus,

$$\begin{array}{c} \bar{R}\approx \frac{3}{4}\bar{S} \end{array}$$ (25)

The mean delay time for a packet from the LQ can be estimated as,

$$\begin{array}{c} \frac{{\bar{W}}_{HQ}}{{\bar{W}}_{LQ}}=1-\rho \end{array}$$ (26)

$$\begin{array}{c} {\bar{W}}_{LQ}=\frac{{\bar{W}}_{HQ}}{1-\rho } \end{array}$$ (27)

Let *W*_*T*_ represent the wasted time between sequent transmissions that is equal to the sum of the gaps between the packets transmissions and the related contention periods in addition to the contention periods in which the channel is not chosen for submission. A uniform distribution is used to provide the fairness property for the channels. Thus,

$$\begin{array}{c} W_{T}=P_{i}\sum_{i=1}^{MaxF_{n}}\left|T_{ci}-T_{tri}\right|+\left(1-P_{i}\right)\left\lfloor \frac{\tau_{off}}{{\bar{T}}_{c}}\right\rfloor {\bar{T}}_{c} \end{array}$$ (28)

where *P*_*i*_ is the probability that the sink node will choose the channel for the next submission and *MaxF*_*n*_ is the maximum number of transmissions that can be achieved in the channel’s idle time, *τ*_*off*_, after deducting the time in which the channel was not chosen for submission, *T*_*cm*_.

$$\begin{array}{c} MaxF_{n}=\frac{\tau_{off}-T_{cm}}{Max(T_{c},T_{tr})} \end{array}$$ (29)

$$\begin{array}{c} W_{T}\approx P_{i}MaxF_{n}\left|T_{ci}-T_{tri}\right|+\left(1-P_{i}\right)\tau_{off} \end{array}$$ (30)

Each channel has the same probability of selection among the *C* channels. Thus,

$$\begin{array}{c} P_{i}=\frac{1}{C} \end{array}$$ (31)

The available transmission time equals, (*τ*_*off*_ − *W*_*T*_) and the achievable number of transmissions in frames is,

$$NT_{tr}=\left\lfloor \frac{\tau_{off}-W_{T}}{T_{tr}}\right\rfloor$$ (32)

The net transmission time is estimated as,

$$\begin{array}{c} NT_{tr}\left(T_{tr}-SIFS-T_{ACK}\right)=NT_{tr}L \end{array}$$ (33)

Considering that the probability of a node to submit in a specific idle time is, *P*_*s*_ and the CC channel’s non-blocking probability is, *P*_*b*_, that can be expressed as $P_{s}=\exp^{-\frac{N}{C}}$ and $P_{b}=1-\frac{{\bar{T}}_{c}}{\bar{S}}$, respectively, the achievable throughput is estimated as,

$$\begin{array}{c} D_{off}=\left(1-P_{s}P_{b}\right)NT_{tr}LD_{lc} \end{array}$$ (34)

Accordingly, the channel’s utilization efficiency will be,

$$\begin{array}{c} \eta =\frac{D_{off}}{\tau_{off}D_{lc}} \end{array}$$ (35)

### Performance Evaluation

**Fig 4:**
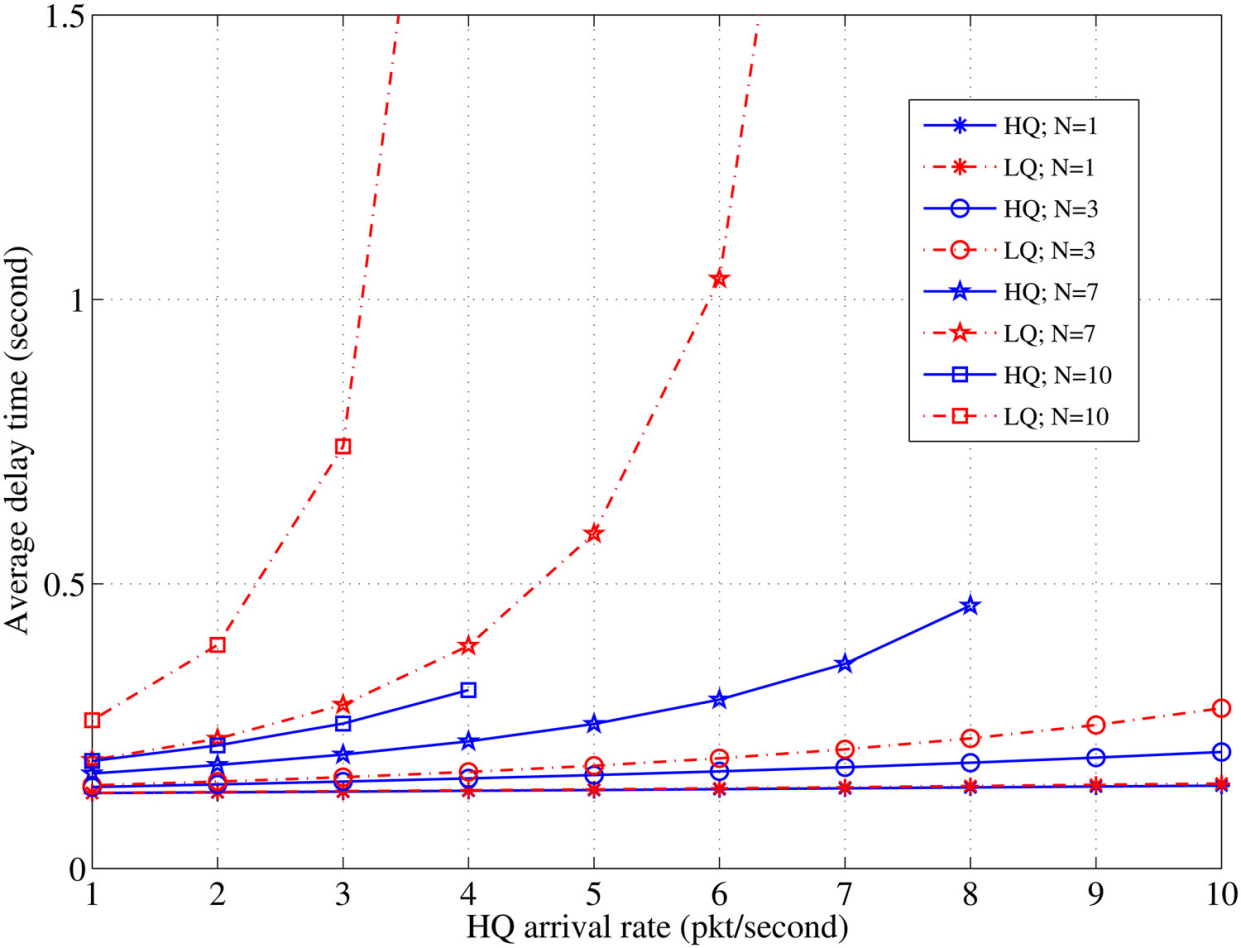
Relationship between *λ*_*HQ*_ and the delay in case of various values of *N*; *τ*_*off*_ = 0.5, *λ*_*LQ*_ = 3 [pkt/sec].

The evaluation results demonstrate the significant impact of the contention time and the packet length on decreasing the induced delay and increasing the efficiency of the channel utilization. The results also provide a set of values for the node density and the number of channels that result in the best performance in each cluster. The evaluation is performed in MATLAB with the general parameters as listed in Table 1. Fig 4 shows the effects of the arrival rates of the high-priority packets on the average delay for different node populations. However, the average delay increases for the HQ and LQ packets as the HQ arrival rate increases; the induced average delay of the LQ packets is more than that of the HQ packets because the HQ packets are prioritized to be sent first. The LQ packets demonstrate a greater sensitivity against the increase in the node density and their average delay curves begin to behave exponentially, faster than those of the HQ packets. Thus, the system becomes unstable at lesser HQ arrival rates, (*λ*_*HQ*_ > 8; *N* = 7) and (*λ*_*HQ*_ > 4; *N* = 10). It can be observed that the node density changes have a more significant effect on the system stability than on the average delay of the packets. This phenomenon occurs because the increase in the node density significantly affects the CC channel utilization.

**Table 1:** Evaluation parameters values.

Parameter	Value
Contention Window minimum size (*CW*_*min*_)	32
Contention Window maximum size (*CW*_*max*_)	1024
Common Channel Data Rate (Mbps)	1
Data Channel Data Rate (Mbps)	1
Slot time in CC Channel (ms)	0.02
*CCA* (ms)	0.02
*DIFS* (ms)	0.05
*SIFS* (ms)	0.01
*T*_*RTS*_ (ms)	0.352
*T*_*CTS*_ (ms)	0.304
*T*_*ACK*_ (ms)	0.304

**Fig 5:**
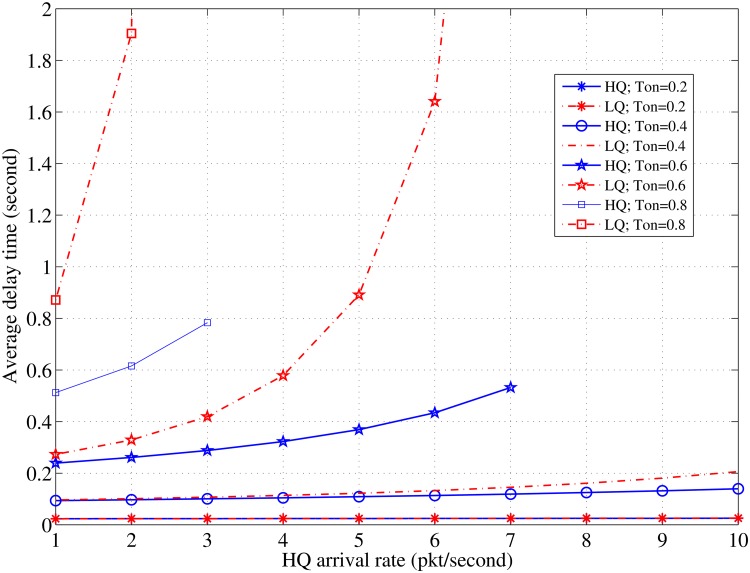
Relationship between *λ*_*HQ*_ and the delay in case of various values of *τ*_*off*_; *N* = 10, *λ*_*LQ*_ = 3 [pkt/s].

Comparison of the results in Figs 4 and 5 illustrates that the effect of the node density is less than the effect of the channel’s idle time length, particularly, at higher packet arrival rates. A long idle time can significantly enhance the average delay even with an increase in the HQ packet arrivals. For instance, when *τ*_*off*_ = 0.8, the average delay does not exceed 0.03 s for both the HQ and the LQ packets. The exponential increases during short periods of the idle time are faster than in denser node populations. Further, the average delay increase induced by the increase in the HQ traffic with various values of the channel idle time is greater than that with various nodes densities. The system sensitivity against instability with shorter idle times is more than that with higher node densities (*λ*_*HQ*_> 7; *τ*_*on*_ = 0.6) and (*λ*_*HQ*_> 3; *τ*_*on*_ = 0.8) with the same number of allocated channels (*C* = 10). That is because the data channel utilization has a more significant effect than the CC channel utilization. While the delay presented in literature [21] shows exponential behavior at lesser PU traffic and high priority traffic, and at a denser PU traffic in a previous study [20], the exponential behavior begins at a denser licensed traffic in the proposed algorithm, as shown in Fig 5.

**Fig 6:**
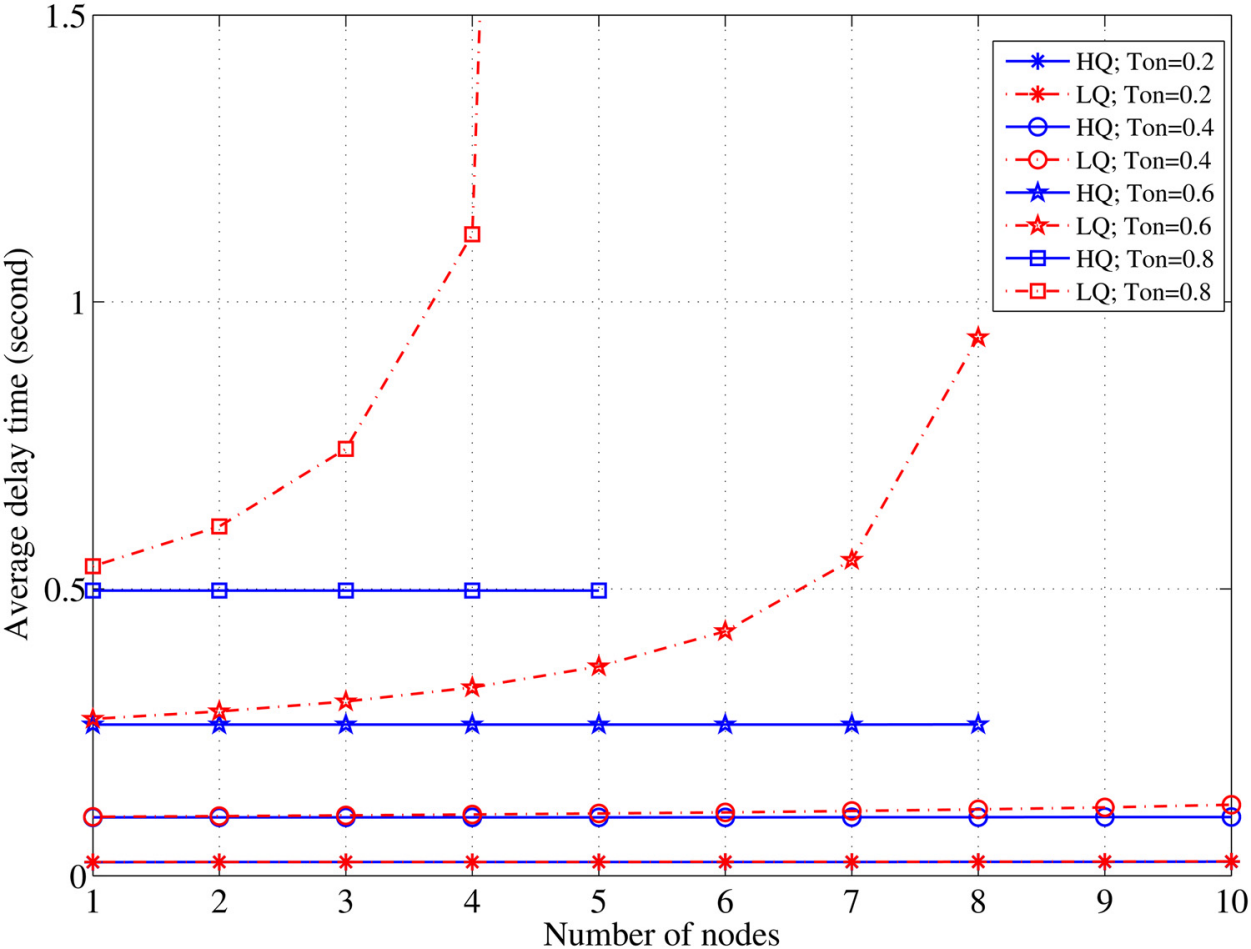
Relationship between *N* and the delay in case of various values of *τ*_*off*_; *λ*_*LQ*_ = *λ*_*HQ*_ = 3 [pkt/s].

Fig 6 reinforces the findings shown in the previous figures, wherein, in a constant period of the channel idle time, the average delay of the HQ packets is marginally affected by the increase in the node density. On the contrary, the average delay of the LQ packets is significantly affected by an increase in *N*. In case of a constant traffic pattern, the *τ*_*off*_ length has the most significant influence on the average delay. These results determine the appropriate parameters for transmitting voice packets within acceptable latency bounds of up to 250 ms.

**Fig 7:**
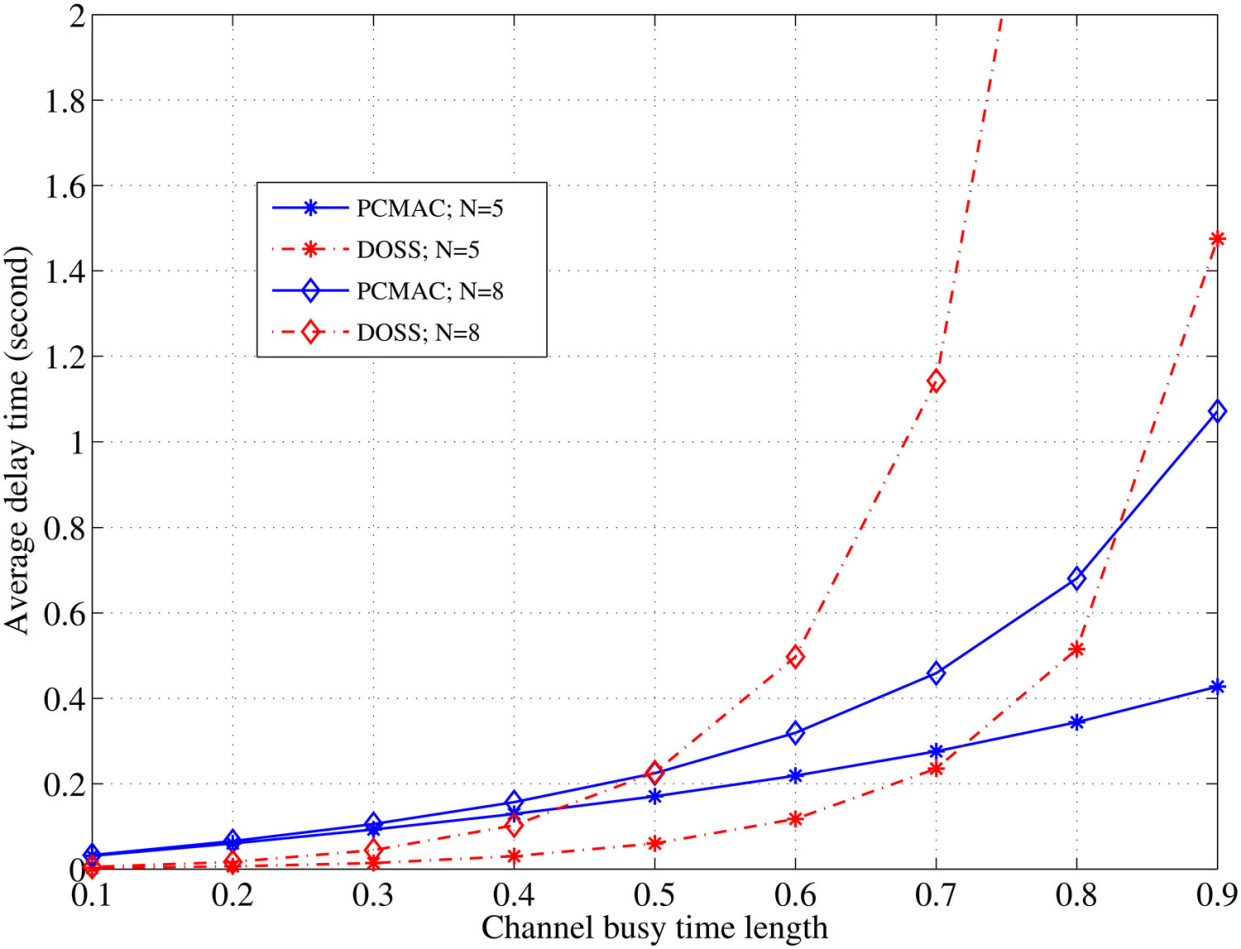
Average delay comparison between PCMAC and DOSS MAC protocols; *C* = 5; *λ*_*LQ*_ = *λ*_*HQ*_ = 2 [pkt/s]; L = 3kb.

Fig 7 shows the performance comparison between the proposed MAC protocol and the DOSS MAC Protocol [20]. Although the DOSS MAC presents a shorter delay time for the SU packets during short lengths of the channel’s busy time, it behaves exponentially in the longer idle times starting from 0.6 s. The PCMAC presents a more stable performance for both the short and long periods of the channel’s idle time even though it is affected by the increase in the node density, N = 8, and begins to behave exponentially. It is obvious that the DOSS protocol is more sensitive against node density changes because it adopts a concurrent transmission through several channels that becomes difficult when more nodes are involved in the contention.

**Fig 8:**
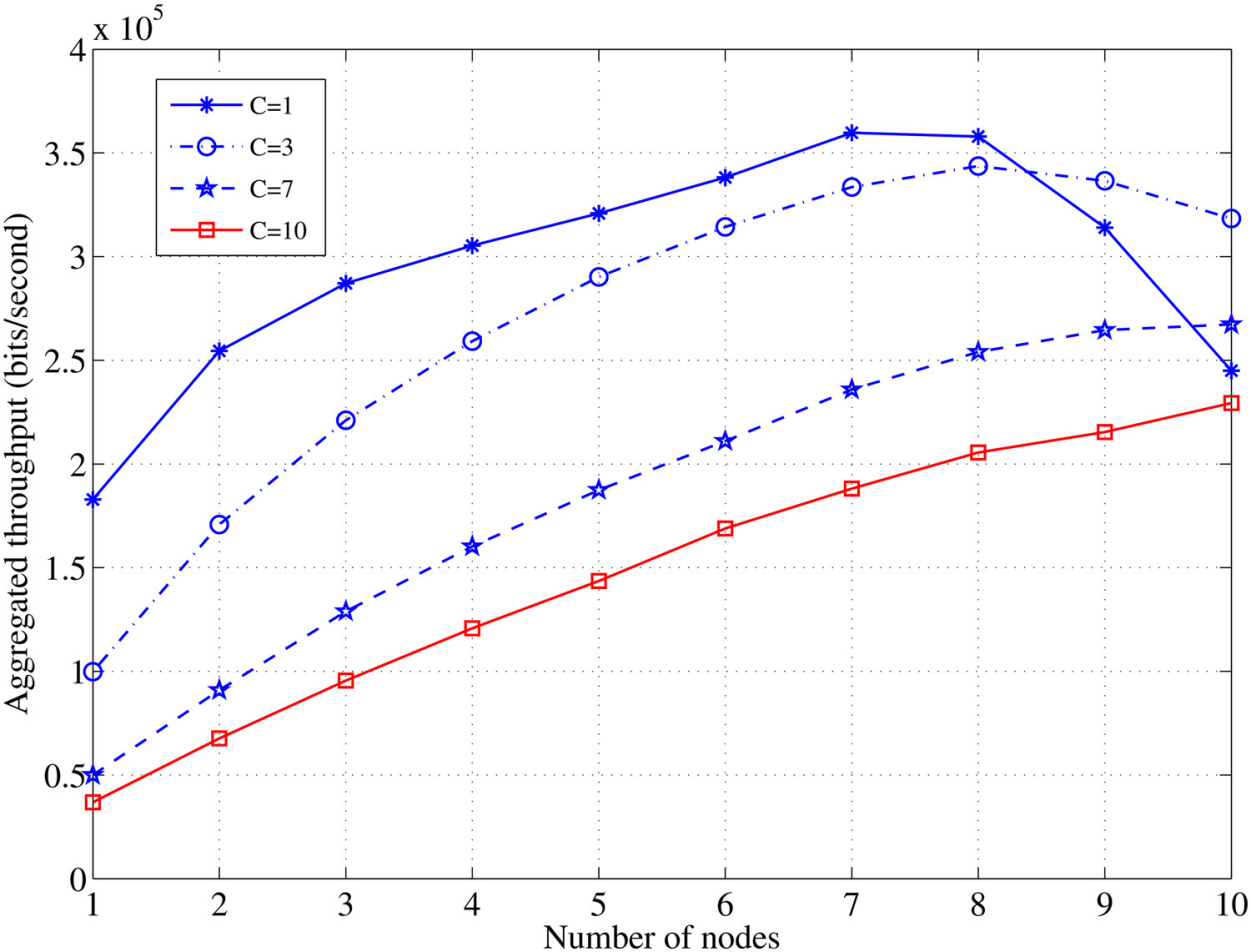
Relationship between *N* and aggregated throughput in case of various values of *C*; *τ*_*off*_ = 0.5, *L* = 1000 [bits].

Fig 8 shows the effects of the node density on the aggregated throughput in the case of various numbers of channels and a fixed length of the channel idle time, *τ*_*off*_ = 0.5. The curves demonstrate that the throughput will increase rapidly with the increase in the node density until they reach their maximum values when *N* = 7 and then they start to slide down. This behavior of the throughput is similar to that in a previous work [28] that describes the relationship between the increase in the node density and the throughput in a CSMA-based common control channel. This trend is very clear with a fewer number of channels and it reaches its highest when *C* = 1; however, it marginally appears with an increase in the available channels. The reason for the change from an increase to a rapid decline is attributed to the value of the time gap between the contention time, *T*_*c*_ and the transmission time, *T*_*tr*_ that decreases as the node density increases until the lowest value, when *N* = 7 is obtained.

**Fig 9:**
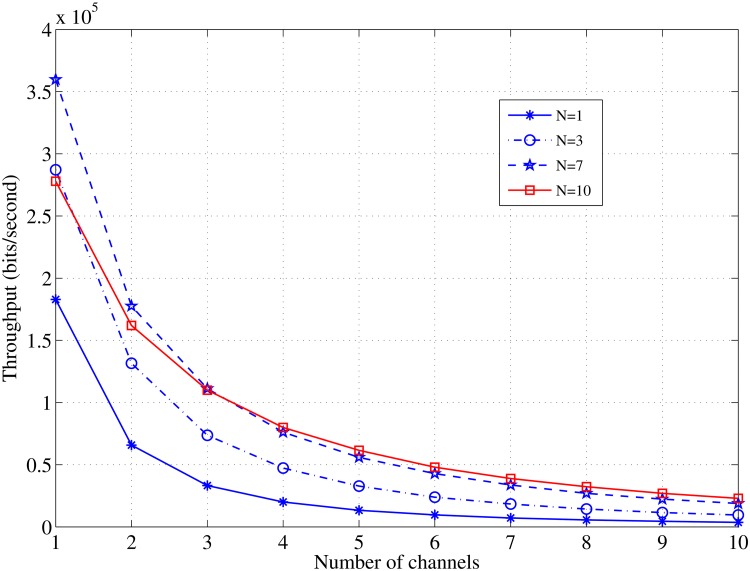
Relationship between *C* and the throughput in case of various values of *N*; *τ*_*off*_ = 0.5, *L* = 1000 [bits].

It can be seen that a large number of the channels do not induce a significant increase in the aggregated throughput. Fig 9 shows the effect of the number of channels on throughput at various nodes densities. It is obvious that the throughput per channel decreases as the available number of channels increase and it is not necessary that a high node density induces a greater throughput; when the number of nodes *N* = 7, a higher value of the throughput per channel is achieved.

**Fig 10:**
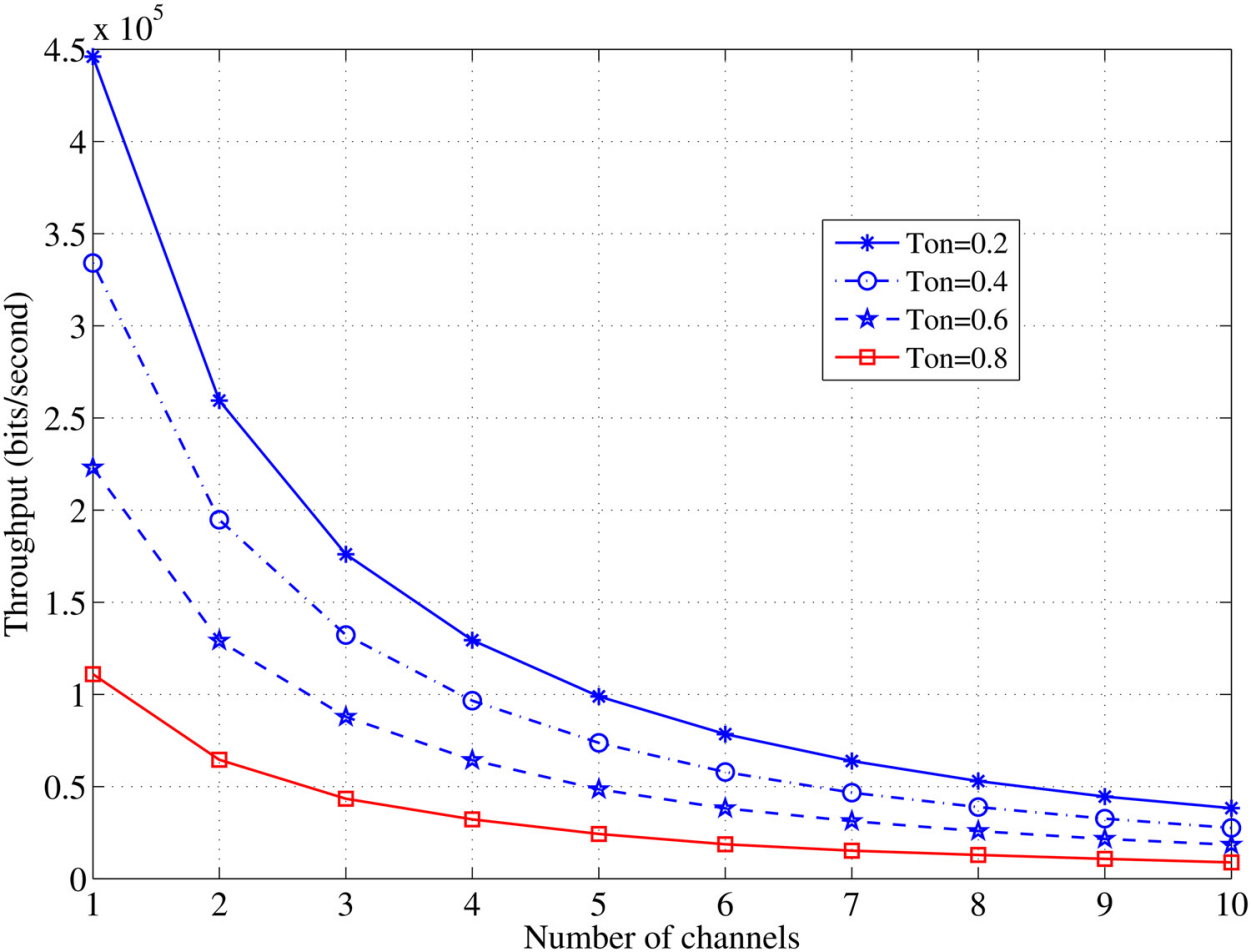
Relationship between *C* and the throughput in case of various values of *τ*_*off*_; *N* = 10, *L* = 1000 [bits].

**Fig 11:**
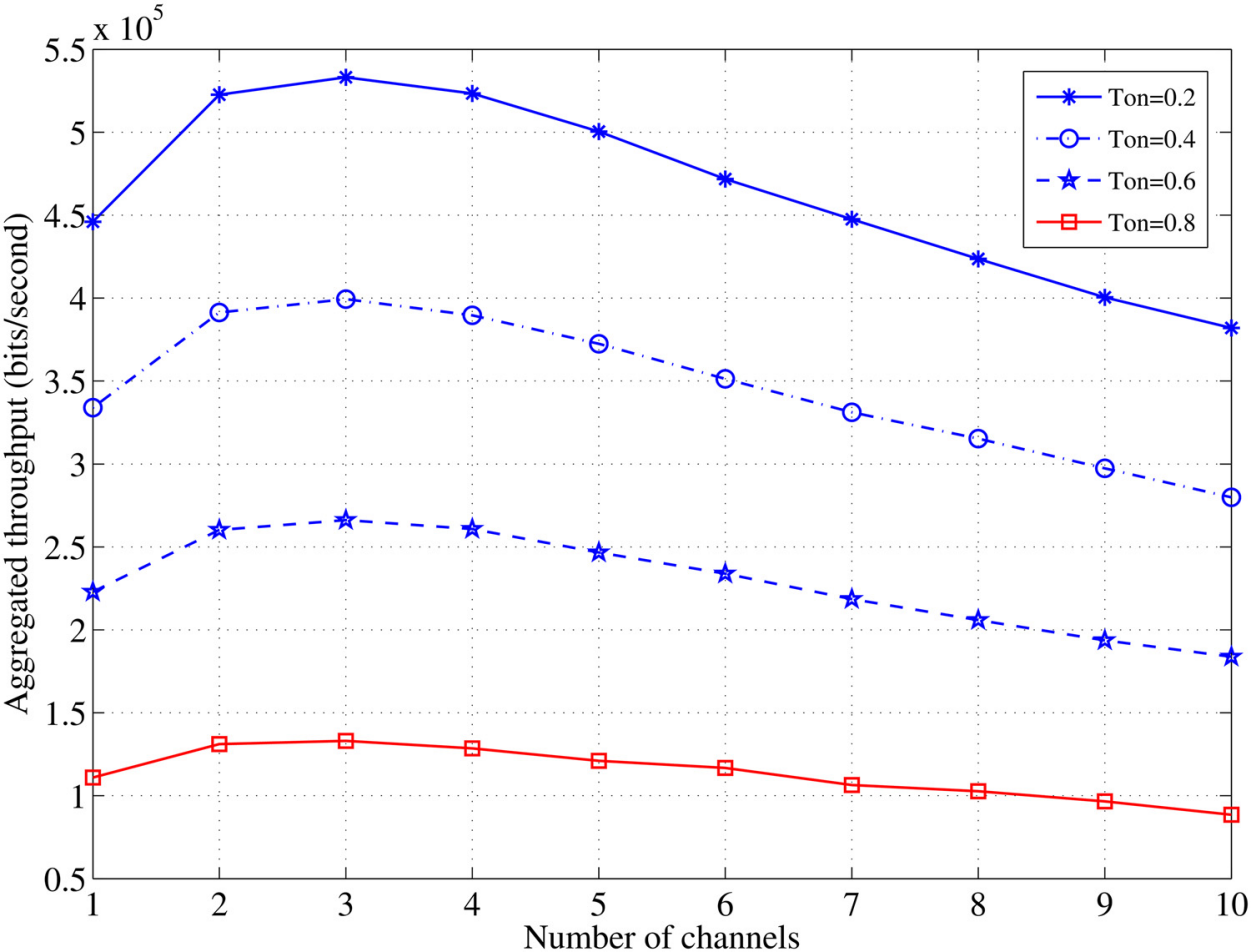
Relationship between *C* and the aggregated throughput in case of various values of *τ*_*off*_; *N* = 10, *L* = 1000 [bits].

The results in Figs 10 and 11, illustrate the significant effect of the idle time on the achieved throughput and the aggregated throughput with the increase in the available channels. The most interesting inference is that increasing the number of the available channels may not significantly increase the throughput, and it is sufficient to allocate a few channels to obtain a satisfactory throughput and an aggregated throughput in the case of a constant channel idle time.

Fig 10 demonstrates the significant effect on the throughput by variations in the channel’s idle time. Moreover, Fig 11 confirms the last aforementioned point and shows that increasing the number of the channels induces a modest increase in the aggregated throughput. A similar throughput behavior was observed in the previous literature [28] and shows that a higher throughput can be achieved with a fewer number of allocated channels. According to the findings of this study and in a homogeneous radio environment, the best performance can be achieved when the master nodes are set as seven nodes each and allocated only two channels.

**Fig 12:**
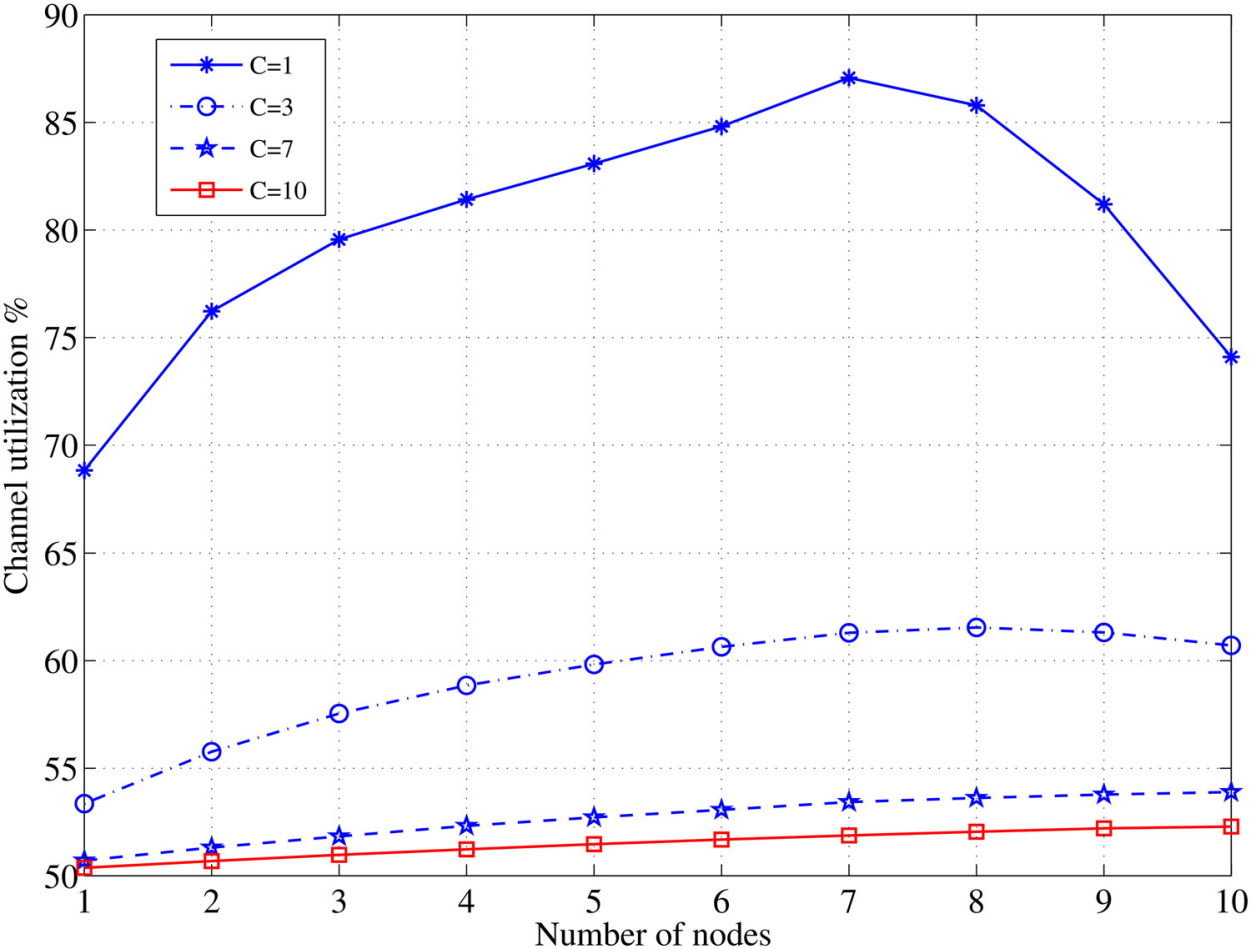
Channel utilization ratio as a function of node density for different values of *C*; *τ*_*off*_ = 0.5, *L* = 1000 [bits].

The channel utilization ratio, *η*, reaches its highest value (87%) when *C* = 1 and *N* = 7, and it decreases as the number of channels increase because the load will be shared between the participating channels, as illustrated in Fig 12. Note that the channel efficiency is estimated regarding the PU’s busy time.

**Fig 13:**
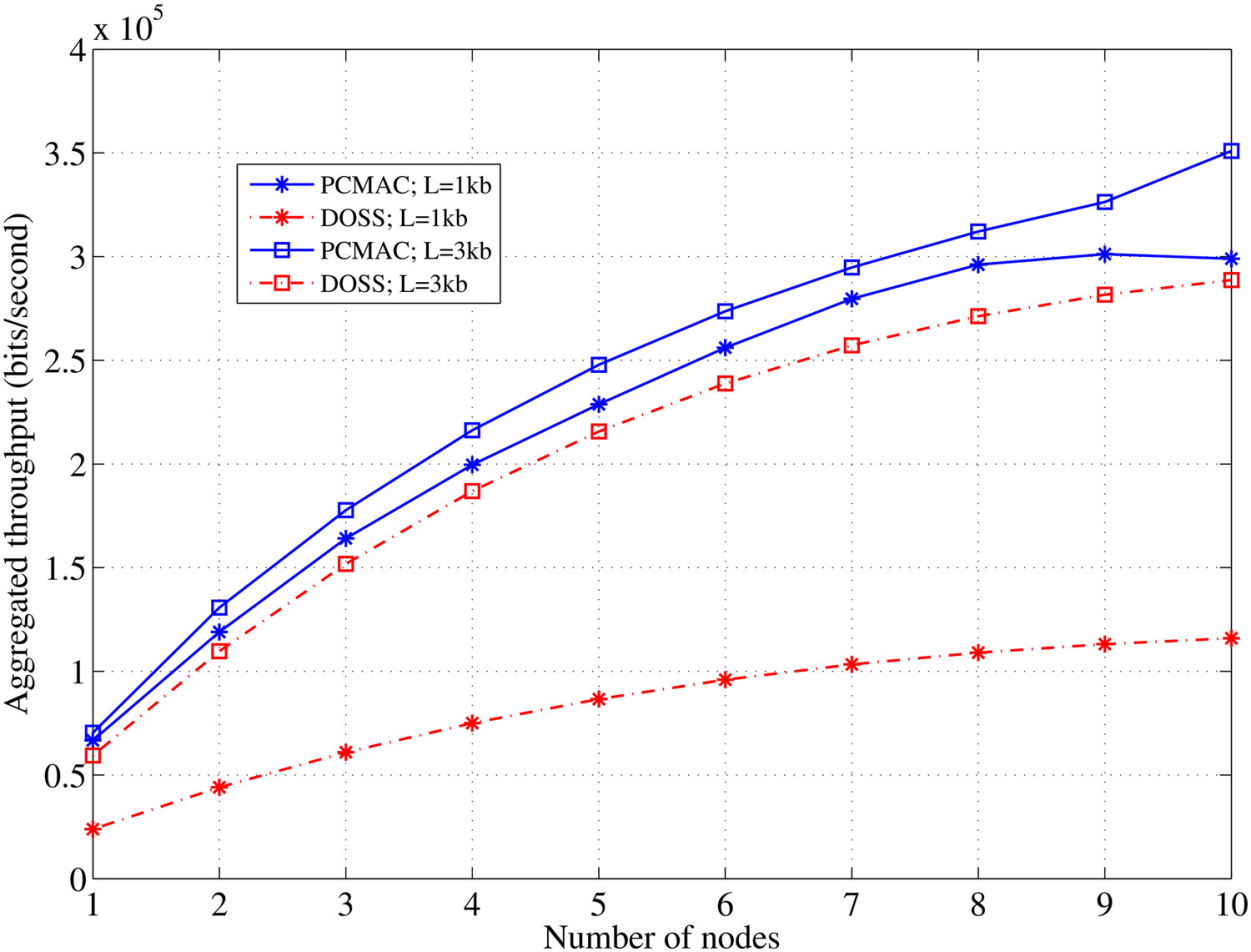
Comparison curves of the aggregated throughput for PCMAC and DOSS MAC in various lengths of the data frame; *τ*_*off*_ = 0.5, *C* = 5.

Because of the burstiness nature of the traffic in the PCMAC protocol, it shows a better performance than the DOSS MAC protocol in terms of the achievable throughput, particularly with short-length packets, e.g. *L* = 1 kb, as demonstrated in Fig 13. Longer packet lengths, e.g. *L* = 3 kb, improve the DOSS MAC protocol’s performance significantly, though the decrease in the node density slows down the increase in the throughput owing to the contention in the CC channel. Simultaneous transmission and concurrent submission through several channels for the packets in the DOSS cause a high sensitivity against the increase in the node density.

## Conclusion

In this study, the design and performance modeling of a new MAC algorithm is proposed that addresses the challenges in the feasible implementation of the CR in wireless sensor applications as well as the emerging application requirements. The PCMAC protocol considers traditional WSN features such as the low complexity and the low power consumption. At the same time, the PCMAC addresses a variety of node traffic criticality in an appropriate scheduling and resource management scheme. Further, the performance evaluation of the proposed algorithm shows a satisfactory performance for heterogeneous traffic, particularly, for real time traffic. The performance is evaluated considering the effects of the different parameters. In addition to highlighting the system instability conditions, the results illustrate the optimal values of these parameters that achieve the best latency performance and an efficient radio resource utilization. The findings highlight that the channel idle time is the key factor for the performance. Moreover, the simulation results prove the robust performance of the proposed protocol compared to similar protocols.

## References

[1] Becker M. Services in Wireless Sensor Networks: Modelling and Optimisation for the Efficient Discovery of Services. Springer Science & Business Media, 2014.

[2] Yang SH. Wireless sensor networks Principles, Design and Applications. Springer London 2014.

[3] Akyildiz I.F., Lee W.Y., Vuran M.C., Mohanty S. NeXt generation/dynamic spectrum access/cognitive radio wireless networks: A survey. Computer Networks. vol: 50 no: 13 pp: 2127–2159. 2006 10.1016/j.comnet.2006.05.001

[4] Zahmati AS, Hussain S, Fernando X, Grami A. Cognitive wireless sensor networks: emerging topics and recent challenges. IEEE Toronto International Conference Science and Technology for Humanity (TIC-STH). pp: 593–596. 2009 10.1109/TIC-STH.2009.5444432

[5] Ghosh M. Cognitive radios in television white spaces Television Goes Digital. pp: 187–207. Springer New York 2009.

[6] Wang XY, Wong A. Multi-Parametric Clustering for Sensor Node Coordination in Cognitive Wireless Sensor Networks. PLoS ONE 8(2): e53434 2013 10.1371/journal.pone.0053434 23418421 PMC3572107

[7] Collotta M, Gentile L, Pau G, Scata G. Flexible IEEE 802.15.4 deadline-aware scheduling for dpcss using priority-based csma-ca. Computers in Industry. vol: 65 no: 8 pp: 1181–1192. 2014 10.1016/j.compind.2014.07.004

[8] IEEE W. Group. IEEE standard for local and metropolitan area networks part 15.4: Low-rate wireless personal area networks (LR-WPANS). IEEE Std. vol: 802 pp: 4–2011. 2011.

[9] Huang P, Xiao L, Soltani S, Mutka MW, Xi N. The evolution of mac protocols in wireless sensor networks: A survey. IEEE Communications Surveys & Tutorial. vol: 15 no: 1 pp: 101–120. 2013 10.1109/SURV.2012.040412.00105

[10] IEEE. S. Association. Part 22: Cognitive wireless ran medium access control (MAC) and physical layer (PHY) specifications: Policies and procedures for operation in the tv bands. IEEE Standard. vol. 802 2011.

[11] Ergul O, Akan OB. Energy-efficient cooperative spectrum sensing for cognitive radio sensor networks. IEEE Symposium on Computers and Communications (ISCC). pp: 465–469. IEEE. 2013.

[12] Ergul O, Akan OB. Cooperative coarse spectrum sensing for cognitive radio sensor networks. IEEE Wireless Communications and Networking Conference (WCNC). pp: 2055–2060. IEEE. 2014 10.1109/WCNC.2014.6952606

[13] Shah G, Akan OB. Cognitive adaptive medium access control in cognitive radio sensor networks. IEEE Transactions on Vehicular Technology. vol: 64 no: 2 pp: 757–767. 2015 10.1109/TVT.2014.2324617

[14] Ma L, Han X, Shen C-C. Dynamic open spectrum sharing MAC protocol for wireless ad hoc networks. First IEEE International Symposium on New Frontiers in Dynamic Spectrum Access Networks. DySPAN. pp: 203–213. IEEE. 2005.

[15] Kim KJ, Kwak KS, Choi BD. Performance analysis of opportunistic spectrum access protocol for multi-channel cognitive radio networks. Journal of Communications and Networks. vol: 15 no: 1 pp: 77–86. 2013 10.1109/JCN.2013.000013

[16] Gulbahar B, Akan OB. Information theoretical optimization gains in energy adaptive data gathering and relaying in cognitive radio sensor networks. IEEE Transactions on Wireless Communications. vol: 11 no: 5 pp: 1788–1796. 2012 10.1109/TWC.2012.030812.111166

[17] Lee W-Y, Akyldiz IF. A spectrum decision framework for cognitive radio networks. IEEE Transactions on Mobile Computing. vol: 10 no: 2 pp: 161–174. 2011.

[18] Mariani A, Giorgetti A, Chiani M. Recent advances on wideband spectrum sensing for cognitive radio Cognitive Communication and Cooperative HetNet Coexistence. pp: 1–31. Springer 2014.

[19] Ghasemi A, Sousa ES. Spectrum sensing in cognitive radio networks: requirements, challenges and design trade-offs. IEEE Communications Magazine. vol.: 46 no: 4 pp: 32–39. 2008 10.1109/MCOM.2008.4481338

[20] Shah G, Akan OB. Performance analysis of csma-based opportunistic medium access protocol in cognitive radio sensor networks. Ad Hoc Networks. vol: 15 pp: 4–13. 2014 10.1016/j.adhoc.2013.03.014

[21] Suliman I, Lehtomaki J. Queueing analysis of opportunistic access in cognitive radios Second International Workshop on Cognitive Radio and Advanced Spectrum Management. CogART 2009. pp: 153–157. IEEE. 2009.

[22] Park J-H, Nam Y, Chung J-M. Analysis of channel access with spectrum handoff in cluster based cognitive radio sensor networks 2013 International Conference on ICT Convergence (ICTC). pp: 232–233. IEEE. 2013.

[23] Deng R, Chen J, Yuen C, Cheng P, Sun Y. Energy-efficient cooperative spectrum sensing by optimal scheduling in sensor-aided cognitive radio networks. IEEE Transactions on Vehicular Technology. vol: 61 no: 2 pp: 716–725. 2012 10.1109/TVT.2011.2179323

[24] Liang Z, Zhao D. Quality of service performance of a cognitive radio sensor network IEEE international conference on Communications (ICC). pp: 1–5. IEEE. 2010.

[25] Abbasi AA, Younis M. A survey on clustering algorithms for wireless sensor networks. Computer communications. 30(14). pp: 2826–2841. 2007 10.1016/j.comcom.2007.05.024

[26] Zhao H, Garcia-Palacios E, Wei J, Xi Y. Accurate available bandwidth estimation in ieee 802.11-based ad hoc networks. Computer Communications. vol: 32 no: 6 pp: 1050–1057. 2009 10.1016/j.comcom.2008.12.031

[27] Bianchi G. Performance analysis of the ieee 802.11 distributed coordination function. IEEE Journal on Selected Areas in Communications. vol: 18 no: 3 pp: 535–547. 2000 10.1109/49.840210

[28] Kang D, Park S, Joo C, Bahk S. Address-free contention in wireless access networks with common control channel for throughput improvement. Computer Networks. vol: 64 pp: 112–124. 2014 10.1016/j.comnet.2014.02.009

